# Characterization and management of facial angiofibroma related to tuberous sclerosis complex in the United States: retrospective analysis of the natural history database

**DOI:** 10.1186/s13023-022-02496-2

**Published:** 2022-09-14

**Authors:** Sreedevi Boggarapu, Steven L. Roberds, JoAnne Nakagawa, Eric Beresford

**Affiliations:** 1 Nobelpharma America, LLC, Bethesda, MD USA; 2 TSC Alliance, Silver Spring, MD USA

**Keywords:** Tuberous sclerosis complex, Facial angiofibroma, Mechanistic target of rapamycin, Topical mTOR inhibitor, Surgical removal

## Abstract

**Background:**

Facial angiofibroma is the most predominant cutaneous manifestation of tuberous sclerosis complex (TSC), a rare autosomal dominant genetic disorder impacting the mechanistic target of rapamycin (mTOR). Facial angiofibroma can bleed spontaneously, impair eyesight, and cause aesthetic disfiguration causing psychological and social stress. To date, there is little or no evidence on the demographics, and other TSC features associated with facial angiofibroma or the use of mTOR inhibitor for the management of facial angiofibroma. This is a retrospective study of TSC Alliance’s Natural History Database aimed to characterize facial angiofibroma and to evaluate features associated with a higher risk of facial angiofibroma or the use of topical mTOR inhibitors for the management of facial angiofibroma. Data in the NHD was obtained from 18 clinical sites in the US since 2006.

**Results:**

Of the 2240 patients, 2088 patients were enrolled in the US and data from 2057 patients were included in this analysis. The mean (median) age of overall TSC patients was 22.4 (19.0) years. A total of 69 patients were ≤ 5 years of age. Facial angiofibroma was noted in 1329 (64.6%) patients with TSC. Patients with facial angiofibroma were older on average (Mean: 25.9 [median, 23.0] vs. 16.0 [12.4 years] years, *p* < 0.0001). In patients with vs. without facial angiofibroma, *TSC2* mutation (38.9% vs. 34.8%) was more common than *TSC1* mutation (12.3% vs. 18.1%), and the incidence rate of most of the other TSC-related manifestations was significantly higher in patients with facial angiofibroma. Majority of patients had focal seizures (72.8% vs. 60.7%), followed by angiomyolipoma (63.7% vs. 21.8%) and renal cysts (59.4% vs. 33.5%). The age groups, 11–17 (odds ratio [OR], 2.53) and 18–45 years (5.98), *TSC2* mutation (1.31), focal seizures (1.50), ADHD (1.47) angiomyolipoma (2.79), and renal cysts (2.63) were significantly associated with a higher risk of facial angiofibroma based on multivariate logistic regression. Abrasive or laser therapy was used by 17.1% and 2.6% patients, respectively. Topical mTOR inhibitor use was noted for 329 (24.8%) patients with facial angiofibroma. Overall systemic mTOR inhibitor use was observed in 399 (30.0%) patients for management of one or more TSC manifestations. Use of systemic mTOR inhibitor for facial angiofibroma was noted for 163 (12.3%) patients, among whom only 9 (0.7%) patients used exclusively for the management of facial angiofibroma. Of the patients with facial angiofibroma, 44.6% did not receive any treatment. Significantly higher use of topical mTOR inhibitor was associated with the 11–17 years age group (OR, 1.67), anxiety (1.57), angiomyolipoma (1.51), and renal cysts (1.33).

**Conclusions:**

The presence of *TSC2* mutations and most other TSC-related manifestations was significantly higher in patients with facial angiofibroma. About one-fourth of patients with facial angiofibroma used a topical mTOR inhibitor and use of systemic mTOR inhibitor for the management of facial angiofibroma or for the other manifestations was noted for 30.0%. About 44.6% of patients did not receive any treatment for the management of facial angiofibroma.

## Introduction

Facial angiofibroma is the most predominant cutaneous manifestation of tuberous sclerosis complex (TSC), occurring in about 75% to 90% of patients [1, 2]. TSC is an autosomal dominant genetic disorder with an estimated incidence of approximately 1 per 5800 live births [3, 4] affecting about 1 million people worldwide and ~50,000 individuals in the United States. Loss-of-function mutations in either the *TSC1* or *TSC2* gene encode dysfunctional hamartin or tuberin, respectively, disrupting the negative regulation of mechanistic target of rapamycin (mTOR) signaling pathway [3]. The chronic activation of mTOR leads to uncontrolled cell proliferation and, consequently, the appearance of hamartomas and hamartias in multiple organs, including the skin, brain, eyes, heart, lungs, and kidneys [5–7].

Facial angiofibromas are multiple small, pinkish, erythematous hamartomas, typically appearing between 2 and 5 years of age and deteriorate with advancing age. Facial angiofibromas are associated with spontaneous bleeding, pain, risk of infection, facial disfigurement and impairment of patient functions such as vision and breathing, which can ultimately diminish patient’s quality of life [2, 3, 5]. Various approaches are available for the treatment of facial angiofibroma related to TSC [8]. Physical removal of facial angiofibroma through surgical dermabrasion, electrocoagulation, excision, curettage, cryosurgery, or laser therapy are effective short-term approaches, but these are associated with pain, hyperpigmentation, scarring, bleeding, the risk for complication, and recurrence of the lesions [9].

The effectiveness of oral mTOR inhibitors for the treatment of TSC-related manifestations is established for subependymal giant cell astrocytoma (SEGA), renal angiomyolipoma, lymphangioleiomyomatosis (LAM), epilepsy, cardiac rhabdomyoma, and skin lesions [10–15]. However, oral mTOR inhibitors are not approved by regulators for treatment of facial angiofibroma as there could be increased risk of systemic side effects associated with an immunosuppressive effect of these drugs. In addition, melanoma or squamous cell carcinoma were reported as less frequent AEs with sirolimus [16]. however, antitumor effect of sirolimus among kidney transplant recipients with previous squamous-cell carcinoma patient was reported [17].

Various topical formulations of the mTOR inhibitor sirolimus (rapamycin) are effective and generally well tolerated for the management of facial angiofibroma based on several short-term studies [18, 19], which was further established over the long term [20, 21]. Hatano et al. reported improvements in health-related quality of life (HRQoL) in patients treated with sirolimus gel for the management of facial angiofibroma [22]. While the evidence and consensus recommendations suggest the use of topical mTOR inhibitors for the management of facial angiofibroma [3], lack of FDA-approved topical therapy at the time of this study meant there was no standardized formulation available, and many individuals did not have access to topical mTOR inhibitor therapy in the US. Here we present results of a retrospective analysis of the TSC Natural History Database characterizing the natural history of facial angiofibroma and understand treatments in practice in the United States.

## Methods

Data for this analysis were obtained from the TSC Natural History Database Project, which was launched in 2006 by the TSC Alliance, in partnership with a network of TSC clinics in the United States. The TSC Natural History Database Project was created to collect medical data on TSC’s clinical features, treatments, and long-term outcomes. Data from individuals diagnosed with TSC based on genetic or clinical criteria [23] were included in this database. Institutional review board approval was obtained for all the 18 TSC participating clinics in the United States.

A computer-generated unique identifier was assigned to each patient. After the confirmation of the TSC diagnosis and informed consent, all data were extracted from clinical charts under the supervision of the principal investigator (TSC site medical director) and entered into a longitudinal database by a research coordinator at that site. Information was updated regularly with new data from follow-up visits, telephone calls, hospitalizations, and diagnostic tests or procedures.

Patient records in the database are updated at least annually and include information about TSC-related care received at the clinics, as well as retrospectively captured information regarding TSC-related care received outside of the clinics. At the time of the analysis, the database housed information regarding demographics, genotypes, clinical features, diagnostics, and treatments for 2240 patients with TSC. Out of 2240 patients enrolled (data extracted in May 2020) from all the participating countries, 2088 patients were from the United States. In this retrospective analysis, after excluding 31 deceased patients, data from 2057 patients from the United States were included.

The demographics and baseline characteristics that include age at the time of data extraction, age groups, age at diagnosis of TSC, age groups at diagnosis of TSC, biological sex, race, *TSC1* and *TSC2* mutations, TSC-related manifestations, and the number of TSC-related manifestations in each patient were analyzed. The age and age at diagnosis were categorized into 0–2, 3–5, 6–7, 8–10, 11–17, 18–45, ≥46 years. Information on the TSC manifestations was available in the NHD for rhabdomyoma, aneurysm, arrhythmia/dysrhythmia, coarctation aorta, valve dysfunction, other cardiovascular disease, gingival fibromas, dental pits, other dental conditions, liver hamartoma, subependymal giant cell astrocytoma (SEGA), infantile spasms, focal seizures, epilepsy, autism, attention deficit hyperactivity disorder (ADHD), depression, anxiety, other psychiatric disorder, retinal hamartoma, LAM, angiomyolipoma, renal cyst, polycystic kidneys, adrenal angiomyolipoma, bone sclerotic foci, chordoma, calvarium sclerosis and thickening, hemi hypertrophy, lipoma, lymphedema, perivascular epithelial cell tumor (PEComa) of uterus or ovary, scoliosis, spleen angiomyolipoma, stomach hamartoma, testicular angiomyolipoma, and other rare conditions.

A subgroup analysis by age groups including data on all the TSC manifestations studied was conducted. The treatments noted for the management of facial angiofibroma include laser therapy, abrasive therapy, topical mTOR inhibitors, systemic mTOR inhibitors, and other treatments for facial angiofibroma. In addition, the use of systemic mTOR inhibitor was noted for other TSC manifestations. The total number of patients who were using systemic mTOR inhibitor for the management of facial angiofibroma or for other manifestations were analyzed. In addition, use of mTOR inhibitors exclusively for the management of facial angiofibroma after excluding the use noted for other manifestations was evaluated. The reasons for the use of mTOR inhibitor was analyzed among patients with facial angiofibroma.

### Statistical analyses

Disease and patient characteristics were summarized using descriptive statistics. The counts and frequency were reported for categorical variables and the mean and standard deviation were reported for continuous variables. The statistical analyses for comparisons were performed using chi-square tests for categorical variables and *t*-tests for continuous variables. The association of demographics and baseline characteristics with facial angiofibroma or with the use of a topical mTOR inhibitor was analyzed by univariate and further by multivariate analysis. The odds ratio (OR) and confidence intervals (CI) are presented. All the demographic parameters (with >5% in both the groups) and all the major TSC manifestations present in the patients (>10% in at least one group) were included in the multivariate logistic regression analysis. The *p*-value ≤ 0.05 was considered statistically significant. Statistical analyses were performed using R studio version 1.1.383.

## Results

### Demographics and baseline characteristics

**Fig. 1:**
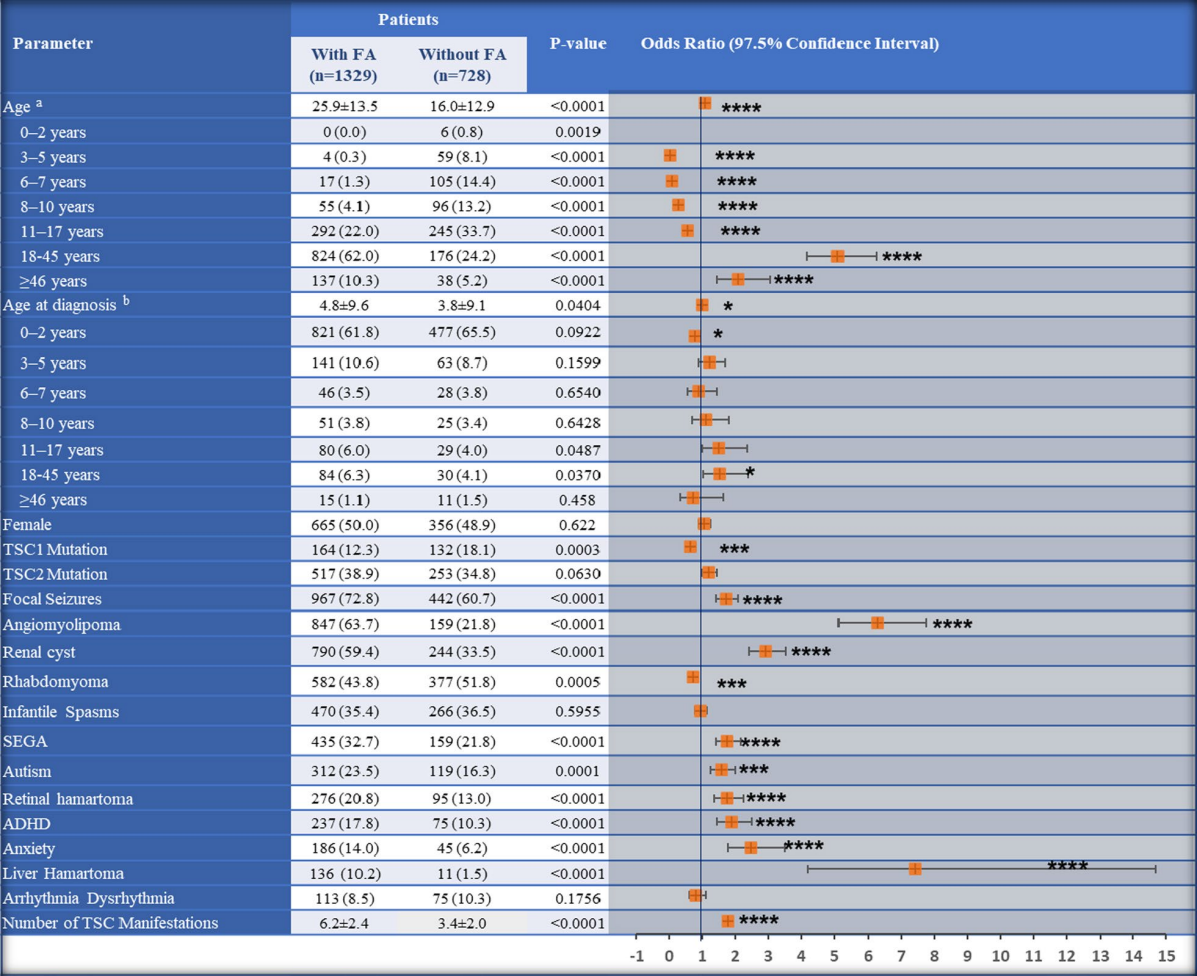
Demographics and baseline characteristics of patients with TSC by the presence of facial angiofibroma. Data are presented as n (%) of patients unless otherwise specified. ^a^Data was available for 2054 (Patients with FA, N = 1329; Patients without FA, N = 725). ^b^Data was available for 1901 (Patients with FA, N = 1238; Patients without FA, N = 663). *p < 0.05; ** < 0.01; ***p ≤ 0.001; ****p < 0.0001. ADHD, attention-deficit/hyperactivity disorder; CI, confidence interval; FA, facial angiofibroma; mTOR, mechanistic target of rapamycin, OR, odds ratio; SD, standard deviation; SEGA, subependymal giant cell astrocytoma; TSC, tuberous sclerosis complex

Of the total 2057 patients with TSC included in this retrospective analysis, 1329 (64.6%) patients had facial angiofibroma. Overall, the mean age (SD) of patients was 22.4 (14.1) years with a median age of 19.0 years. Patients with facial angiofibroma versus without facial angiofibroma were significantly older (25.9 [median, 23.0 years] versus 16.0 [12.4 years] years, *p*<0.0001) on average. The frequency of facial angiofibroma increased with age until 18–45 years, and the frequency was much larger compared to the 11–17 years group. The majority of patients with facial angiofibroma (62.0%) were in the 18–45 years age group, while the majority of patients without facial angiofibroma (33.7%) were in the 11–17 years age group (Fig. 1). The mean age of diagnosis of TSC was significantly higher in patients with facial angiofibroma (4.8 versus 3.8 years, *p*=0.045; Fig 1). The majority of patients were diagnosed with TSC within 2 years of age in both groups (Fig. 1). Very few patients were diagnosed with TSC at ≥46 years of age (patients with facial angiofibroma, 15 [1.1%]; patients without facial angiofibroma, 11 [1.5%]).

The frequency of women and men is similar in both groups (Fig. 1). Overall, the presence of *TSC2* mutation (38.9% vs. 34.8%) was more common than *TSC1* mutation (12.3% vs. 18.1%) in both groups (Fig. 1).

### TSC-related manifestations

The clinical presentation of TSC varied widely between patients with and without facial angiofibroma. The presence of most of the TSC-related manifestations was significantly higher in patients with facial angiofibroma (Fig. 1).

The number of TSC-related manifestations observed in this population varied widely, ranging from 0 to 14 manifestations in any given patient with TSC. Patients with facial angiofibroma showed a significantly higher mean number of TSC manifestations than that observed in patients without facial angiofibroma (6.2 vs. 3.4, *p*<0.0001; Fig. 1).

**Fig. 2:**
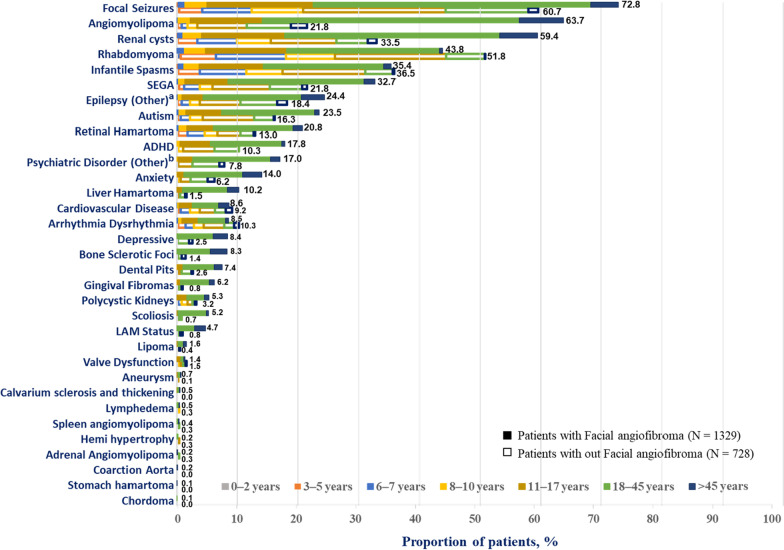
Proportion of patients with TSC manifestations in patients with facial angiofibroma versus without facial angiofibroma by age subgroups. ADHD, attention-deficit/hyperactivity disorder; LAM, lymphangioleiomyomatosis; SEGA, subependymal giant cell astrocytoma; TSC, tuberous sclerosis complex. ^a^Epilepsy (other) such as atonic seizures, clonic seizures, febrile seizures, gelastic seizures etc. ^b^Psychiatric disorder (other) include disorders such as behavioral disorder, aggressive behaviour, anorexia nervosa, learning disorder, mood disorder etc.

TSC-manifestations were further analyzed by age groups (Fig. 2). Focal seizures (72.8% vs. 60.7%) were observed in majority of patients followed by angiomyolipoma (63.7% vs. 21.8%), renal cysts (59.4% vs. 33.5%), and rhabdomyoma (43.8% vs. 51.8%) etc. These manifestations were observed from 3 to 5 years age group and increased with increasing age until 18–45 years (Fig. 2). The age group, 0–2 years was observed among patients without facial angiofibroma. Manifestations such as arrythmia dysrhythmia, cardiovascular disease, retinal hamartoma, SEGA, infantile spasms, rhabdomyoma and focal seizures were observed as early as ≤2 years of age (Fig. 2).

### Patient features associated with facial angiofibroma

The univariate regression analysis showed significantly higher risk of facial angiofibroma with the age groups 18–45 years (OR, 5.09) and ≥46 years (2.08), age group at diagnosis 18–45 years (1.54), the presence of TSC-related manifestations such as focal seizures (1.73), angiomyolipoma (6.29), renal cysts (2.91), SEGA (1.74), autism (1.57), retinal hamartoma (1.75), ADHD (1.89), anxiety (2.47), and liver hamartoma (7.43), and the number of coexisting comorbid conditions (1.77; Fig.. 1). In the multivariate regression, a significantly higher risk of facial angiofibroma was observed for the 11–17 years age group (OR, 2.53), the 18–45 years age group (5.98), *TSC2* mutation (1.31), focal seizures (1.50), ADHD (1.47), angiomyolipoma (2.79), and renal cysts (2.63) (Table 1). The risk of facial angiofibroma doubled from 11–17 years to 18–45 years age groups (Table 1).

**Table 1:** Features associated with significantly higher or lower risk of facial angiofibroma based on multivariate logistic regression

Parameter	Odds ratio (95% CI)	*P* value
11–17 years age group	2.53 (1.72–3.74)	< 0.0001
18–45 years age group	5.98 (3.39–10.61)	< 0.0001
*TSC2* mutation	1.31 (1.01–1.71)	0.0373
Focal seizures	1.50 (1.15–1.95)	0.0024
ADHD	1.47 (1.05–2.08)	0.0250
Angiomyolipoma	2.79 (2.17–3.60)	< 0.0001
Renal cysts	2.63 (2.04–3.39)	< 0.0001

Data included for multivariate analysis was from 1901 patients (Patients with facial angiofibroma, N = 1238; Patients without facial angiofibroma, N = 663)

*ADHD* attention deficit hyperactivity disorder; *CI* confidence interval; *TSC* tuberous sclerosis complex

### Treatments

**Fig. 3:**
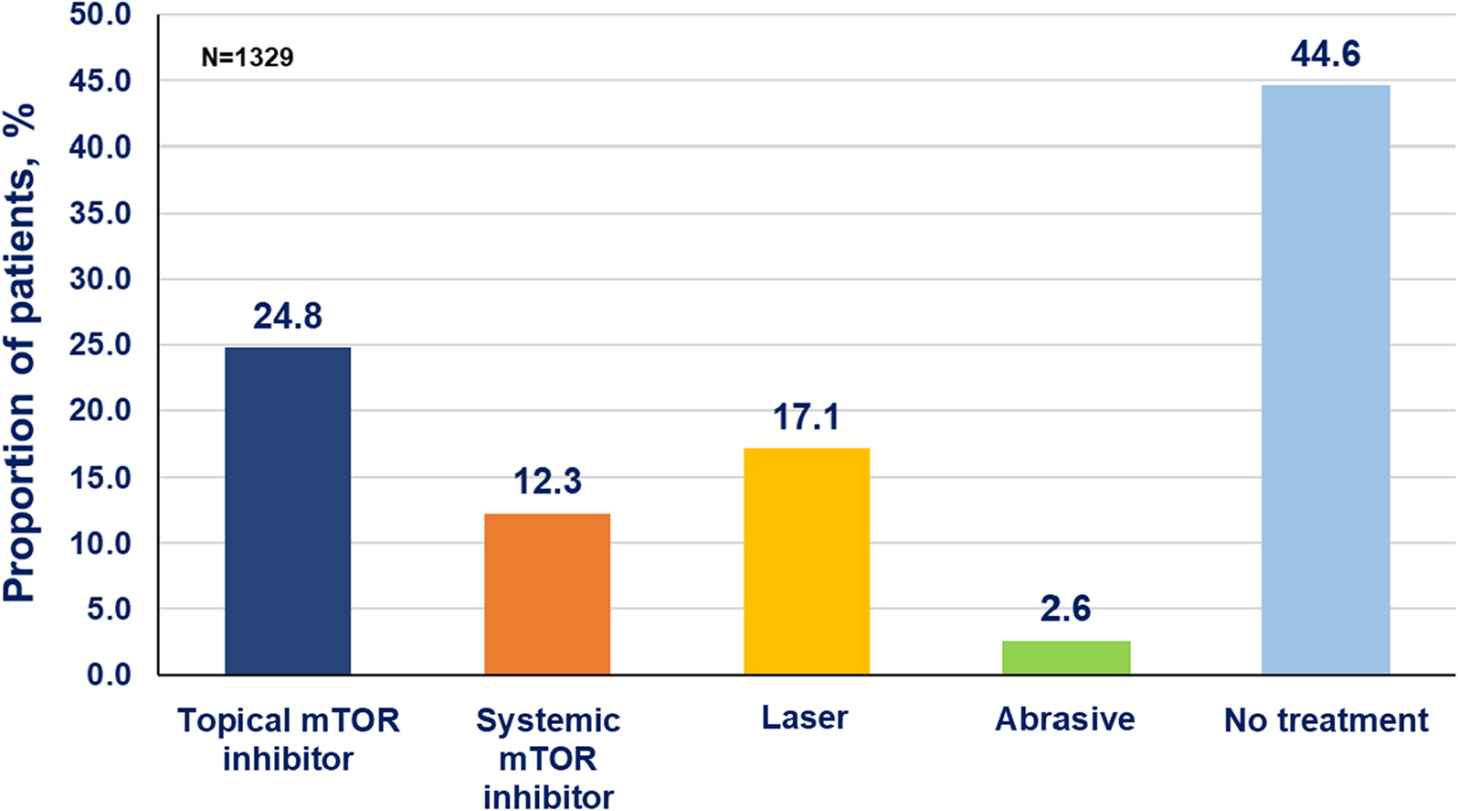
Treatments observed for the management of facial angiofibroma in the Natural history database

Physical removal of facial angiofibroma using laser therapy was noted for 17.1% of patients and abrasive therapy was noted for 2.6% of patients (Fig. 3).

Topical mTOR inhibitor was used by 329 (24.8%) patients for the management of facial angiofibroma (Fig. 3). A total of 593 (44.6%) patients were not receiving any treatment for the management of facial angiofibroma or noted for systemic mTOR inhibitor use for facial angiofibroma or other TSC manifestations (Fig. 3).

Of the patients with facial angiofibroma, 399 (30.0%) patients received systemic mTOR inhibitors for the management of facial angiofibroma or for other TSC manifestations. A total of 163 (12.3%) patients received an mTOR inhibitor for the management of facial angiofibroma (Fig. 3), however, only 9 (0.7%) patients received systemic mTOR inhibitors exclusively for the management of facial angiofibroma.

**Fig. 4:**
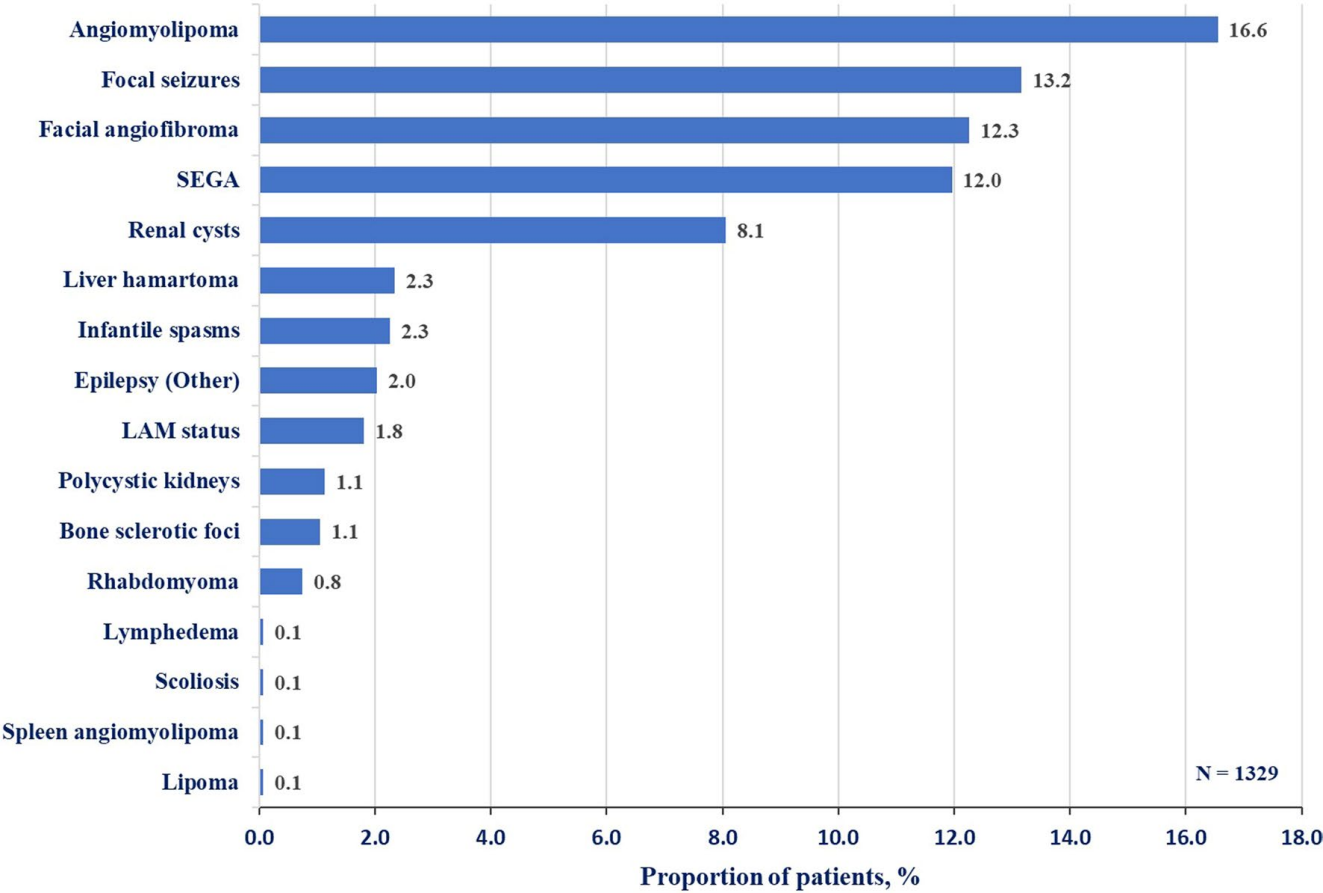
Reason for the use of systemic mTOR inhibitor among patients with facial angiofibroma. mTOR, mechanistic target of rapamycin; LAM, lymphangioleiomyomatosis; SEGA, subependymal giant cell astrocytoma

The proportion of patients with facial angiofibroma who were receiving mTOR inhibitor for other manifestations are presented in Fig. 4. The highest use of systemic mTOR inhibitor was observed for angiomyolipoma (16.4%) followed by focal seizures (13.1%).

### Patient features associated with use of topical mTOR inhibitors

**Fig. 5:**
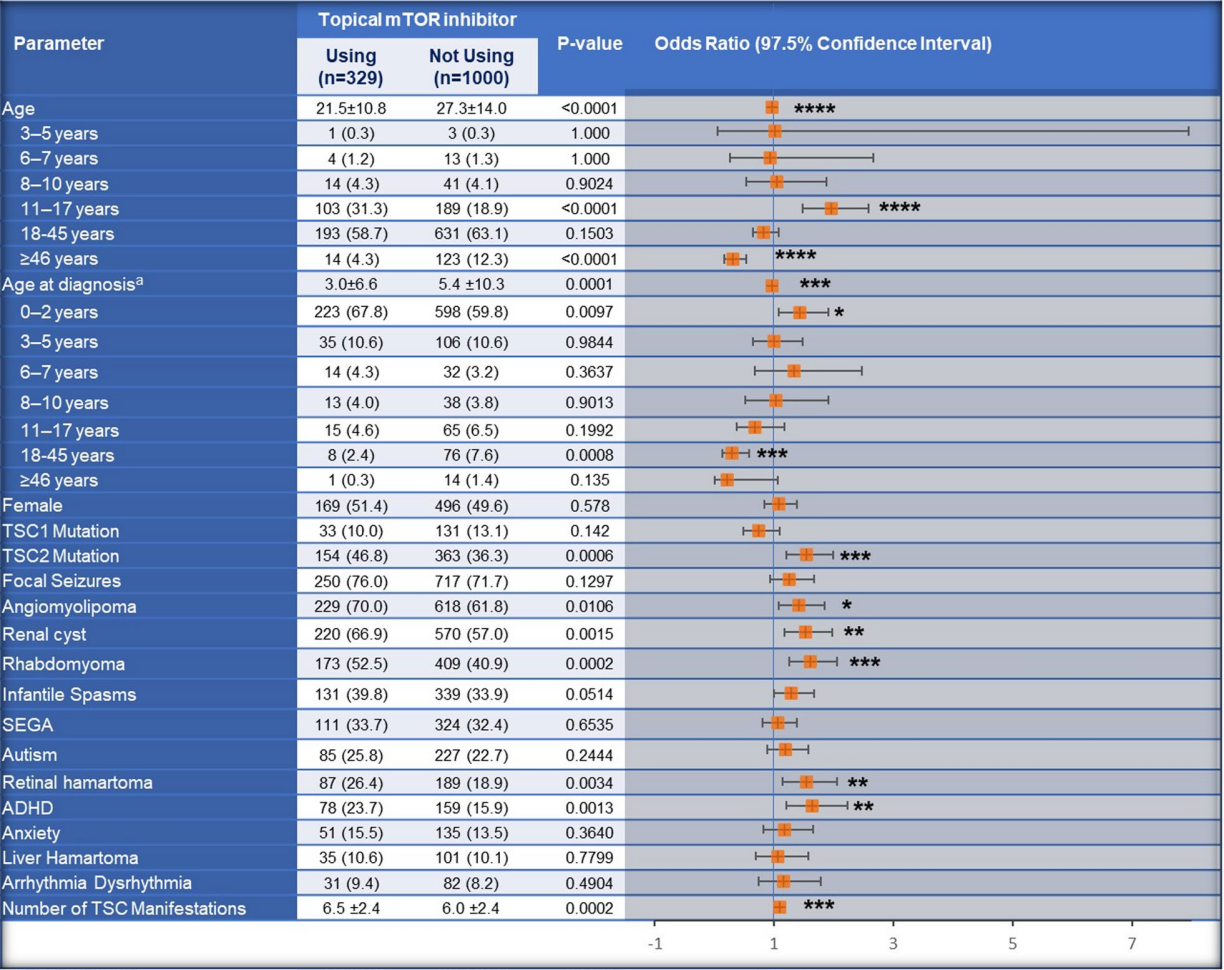
Demographics and baseline characteristics of patients with facial angiofibroma by the use of topical mTOR inhibitors. ^a^Data was available for 1238 patients (Patients using topical mTOR inhibitor, N = 309; Patients not using topical mTOR inhibitor, N = 929). *p < 0.05; ** < 0.01; ***p ≤ 0.001; ****p < 0.0001. ADHD, Attention-deficit/hyperactivity disorder; CI, confidence interval; FA, facial angiofibroma; mTOR, mechanistic target of rapamycin, OR, odds ratio; SD, standard deviation; SEGA, subependymal giant cell astrocytoma; TSC, tuberous sclerosis complex

Topical mTOR inhibitor use was most commonly associated with the 11–17-years age group (OR, 1.96), the 0–2 years age group at diagnosis (1.44), *TSC2* gene mutation (1.54), and the presence of manifestations such as renal cysts (1.52), angiomyolipoma (1.42), rhabdomyoma (1.60), retinal hamartoma (1.54), and ADHD (1.64) based on the univariate regression (Fig. 5). In the multivariate regression, the 11–17 years age group (1.67) and presence of anxiety (1.57), angiomyolipoma (1.51), and renal cysts (1.33) were significantly associated with the higher use of topical mTOR inhibitors (Table 2).

**Table 2:** Patient characters significantly associated with higher or lower use of topical mTOR inhibitors based on the multivariate logistic regression

Parameter	Odds ratio (95% CI)	
11–17 years age group	1.67 (1.01–2.79)	0.0473
Anxiety	1.57 (1.05–2.32)	0.0240
Angiomyolipoma	1.51 (1.12–2.04)	0.0070
Renal cysts	1.33 (1.00–1.77)	0.0477

Data included in the multivariate analysis was from 1238 patients (Patients using topical mTOR inhibitor, N = 309; Patients not using topical mTOR inhibitor, N = 926)

*CI* confidence interval; *mTOR* mechanistic target of rapamycin

## Discussion

This retrospective analysis of the TSC Natural History Database was aimed to characterize facial angiofibroma and to understand the real-world use of treatments in the United States. The presence of facial angiofibroma was increased with age (until 18–45 years). Patients with versus without facial angiofibroma were older on average (Median age, 23.0 years versus 12.4 years). Majority of patients were diagnosed within 2 years of age. The significantly higher risk of facial angiofibroma almost doubled from 11–17 to 18–45 years age groups. The presence of most of the other TSC-related manifestations was significantly higher in patients with facial angiofibroma in this study with focal seizures being the most common in both the groups. The most common TSC manifestations were observed as early as ≤5 years age and the incidence rate increased with increasing age groups until 18–45 years. Manifestations such as cardiovascular disease and retinal hamartoma were observed as early as ≤2 years. Presence of focal seizures, ADHD, angiomyolipoma, or renal cysts were associated with significantly higher risk of facial angiofibroma. In this population recruited from TSC Clinics at major medical centers, 44.6% of patients did not receive any treatment for the management of facial angiofibroma. It is not known whether this gap in treatment is due to low medical need (eg. very small and mild angiofibroma) or lack of access to a safe and effective therapy. Topical mTOR inhibitor use was noted for one-fourth of patients with facial angiofibroma. Overall, 30.0% of patients received systemic inhibitors for the management of facial angiofibroma or other manifestations. The major reason for receiving systemic mTOR inhibitor in patients with facial angiofibroma was renal angiomyolipoma.

Although typically diagnosed in children and infants, TSC can present at any age [24]. Data from TOSCA showed that facial angiofibroma typically appears in childhood and the prevalence continues to increase into adulthood [25]. As expected, the presence of facial angiofibroma increased with age in our study. Although the odds ratio for presence of facial angiofibroma in the ≥46 years age group was numerically less than the odds ratio of the 18–45 years age group, the ≥46 years age group is still at high risk of facial angiofibroma, and the numerical difference may or may not be clinically meaningful.

TSC is a rare disease with various manifestations that can predominantly involve the skin, the central nervous system, heart, kidneys, lungs, and less frequently, the retina, gingiva, and other abdominal organs [23, 26]. The clinical presentation varies largely ranging from mild dermatological findings to severe neurologic morbidity [26]. In our study, we found that patients with facial angiofibroma had a higher burden of most of the TSC manifestations compared with patients without facial angiofibroma. The number of manifestations present in an individual also varied ranging from 0 to14 with an average number of TSC-manifestations being significantly higher in patients with facial angiofibroma. The most frequent TSC-related manifestation was focal seizures in patients with and without facial angiofibroma. However, a significant association with facial angiofibroma was observed for liver hamartoma, followed by angiomyolipoma, renal cysts, and anxiety in the univariate regression. This observation is of particular importance because these manifestations have no externally visible physical signs. Because findings of angiofibroma may precede many potentially severe internal manifestations, accurate and timely diagnosis of angiofibroma could be important for informing clinical surveillance of individuals with TSC.

Management of TSC requires a multidisciplinary approach involving dermatologists, neurologists, pulmonologists, urologists, cardiovascular specialists, pediatricians, and geneticists. Skin lesions are among the most frequent TSC manifestations and one of the major reasons for seeking medical attention [2]. The association of many other TSC-related manifestations in patients with facial angiofibroma illustrates the importance of accurate diagnosis by dermatologists and referral to a comprehensive multi-disciplinary TSC Clinic for surveillance and management of other TSC manifestations.

Facial angiofibroma requires chronic treatment throughout life. The updated International TSC Consensus Group recommendations mention that more evidence is necessary to guide the choice of treatment for TSC-related skin lesions while reviewing existing evidence [27]. Current recommendations also acknowledge the 2018 regulatory approval of topical sirolimus gel 0.2% for facial angiofibroma in Japan [27]. Consensus recommendations call for topical interventions as a first line for flat to moderately raised lesions; surgical interventions are recommended for lesions that do not respond, are more protuberant, or require immediate intervention [27]. The International TSC Consensus Group does not recommend systemic therapy for the exclusive treatment of facial angiofibroma due to the risk of systemic side effects and has found insufficient evidence regarding an additive effect of topical mTOR inhibitors for facial angiofibroma as an adjunct to systemic therapy [27].

Physical removal of facial angiofibroma is painful; often requires anesthesia, which can lead to hyperpigmentation or scarring; and carry a risk of posttreatment infection [9]. In addition, physical removal does not address the underlying cause of TSC, and facial angiofibroma can recur. In this study, 2.6% and 17.1% of patients undergone abrasive and laser therapy, respectively.

Studies with oral mTOR inhibitors showed concurrent improvement in facial angiofibroma and other TSC skin lesions [28–31]. However, side effects associated with systemic exposure to mTOR inhibitors may not justify its use solely for the treatment of facial angiofibroma in most cases. In addition, Kitayama et al. reported that topical application, particularly gel formulation, showed more efficient drug delivery to the skin than the oral sirolimus in a hairless mice study [32].

Topical formulations of mTOR inhibitors have the potential to improve facial angiofibroma related to TSC without systemic exposure and associated side effects. In 2010, Haemel et al. [33] reported the improvement in facial angiofibroma with topical sirolimus treatment for the first time. The effectiveness and safety of topical mTOR inhibitors were further established in several studies [18–20, 34–36]. including the long-term safety and sustained efficacy over 52 weeks [37]. A systematic review and meta-analysis by Leducq et al. concluded that topical mTOR inhibitors (primarily sirolimus) were effective in 95% (115 of 121) of patients for the management of facial angiofibroma [36]. The most frequent regimen observed in the systematic review and metanalysis was 0.1% sirolimus, with 1 or 2 applications per day for 12 weeks . Based on these positive reports, many physicians have been prescribing “off-label” topical sirolimus made by compounding pharmacies [26]. The effectiveness and safety of topical sirolimus are further established in a recent systematic review by Cortell Fuster et al. [38]. Most of the improvement in facial angiofibroma with topical sirolimus occurred during the first month of treatment [18]. In a study by Okanishi et al. early sirolimus gel intervention was effective for the treatment of facial angiofibroma and fibrous plaques and reported the potential to maintain the skin at near-normal levels in patients with TSC [39]. In the Natural History Database, 44.6% of patients with facial angiofibroma did not receive any treatment for the management of facial angiofibroma, and about one-fourth of patients were treated with off-label, compounded topical mTOR inhibitors for the management of facial angiofibroma.

Based on the strong evidence for the effectiveness and safety of topical mTOR inhibitors in the management of facial angiofibroma, topical sirolimus is recently approved by the FDA for the management of facial angiofibroma in the United States. Despite the unavailability of approved topical formulation at the time of the study by Crall et al. topical mTOR inhibitors were used by 47.3% patients for the management of facial angiofibroma. Of these patients, 47.7% had difficulties with insurance coverage [40]. In addition, due to their compounded production, these treatments currently vary widely in dosage (0.03–1.00%), excipients, quality control, and cost [36, 41].

The major limitation of this study is being a retrospective analysis rather than a prospective collection of data. The study was limited to 18 participating TSC Clinics associated with major medical centers; patients managed locally or at other centers may have different characteristics. Also, this study does not include treatment outcomes, patient-reported outcomes such as impact on quality of life or patient and caregiver perceptions of benefits and risks.

## Conclusions

Skin lesions are among the most frequent TSC manifestations and a major reason for seeking medical attention. Despite this, 44.6% of patients with facial angiofibroma did not receive treatment for it. Approximately one-fourth used off-label, compounded topical mTOR inhibitor formulations, and < 20% received laser or abrasive treatments. Notably, the burden of most TSC-related manifestations was significantly higher in patients with facial angiofibroma, illustrating the importance of accurate diagnosis by dermatologists and referral to a comprehensive multi-disciplinary TSC Clinic for surveillance and management of other TSC manifestations.

## Data Availability

The datasets used and/or analyzed during the current study are available from the corresponding author on reasonable request.
